# Association between vitamin D deficiency and vasovagal syncope: A systematic review and meta‐analysis

**DOI:** 10.1002/clc.24035

**Published:** 2023-05-24

**Authors:** Amirmohammad Khalaji, Amir Hossein Behnoush, Masih Tajdini

**Affiliations:** ^1^ Tehran Heart Center, Cardiovascular Diseases Research Institute Tehran University of Medical Sciences Tehran Iran; ^2^ Non–Communicable Diseases Research Center, Endocrinology and Metabolism Population Sciences Institute Tehran University of Medical Sciences Tehran Iran; ^3^ School of Medicine Tehran University of Medical Sciences Tehran Iran

**Keywords:** meta‐analysis, syncope, systematic review, vasovagal syncope, vitamin D

## Abstract

Vasovagal syncope (VVS) is the most prevalent type of syncope and its management includes pharmacologic and non‐pharmacologic interventions. Recently, studies have investigated vitamin D levels in VVS patients. In this systematic review and meta‐analysis, we aim to review these studies to find possible associations between vitamin D deficiency and vitamin D levels with VVS. International databases including Scopus, Web of Science, PubMed, and Embase were searched with keywords related to “vasovagal syncope” and “vitamin D.” Studies were screened and the data were extracted from them. Random‐effect meta‐analysis was conducted to calculate the standardized mean difference (SMD) and 95% confidence interval (CI) for vitamin D levels in comparison to VVS patients and controls. Also, VVS occurrence was measured and the odds ratio (OR) and 95% CI were calculated for comparison of vitamin D deficient cases and nondeficient individuals. Six studies were included with 954 cases investigated. Meta‐analysis showed that patients with VVS had significantly lower vitamin D serum levels in comparison to non‐VVS cases (SMD −1.05, 95% CI −1.54 to −0.57, *p*‐value < .01). Moreover, VVS occurrence was higher in vitamin D‐deficient individuals (OR 5.43, 95% CI 2.40 to 12.27, *p*‐value < .01). Our findings which show lower vitamin D levels in VVS patients can have clinical implications in order for clinicians to pay attention to this when approaching VVS. Further randomized controlled trials are certainly warranted to assess the role of vitamin D supplementation in individuals with VVS.

## Graphical abstract

Studies have investigated vitamin D levels in patients with vasovagal syncope (VVS) and compared them with healthy controls. Our systematic review showed that patients with VVS have significantly lower levels of vitamin D. Also, VVS occurrence was significantly higher in vitamin D‐deficient patients.

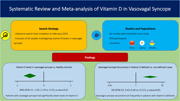

## 1 INTRODUCTION

Syncope is a common chief complaint in patients visiting emergency departments, mostly in women with a peak age of 15 years.^1^, ^2^ Syncope is divided into three categories based on the cause of occurrence—reflex (neurally mediated) syncope, syncope due to orthostatic hypotension, and cardiac syncope.^3^ Vasovagal syncope (VVS), the most prevalent type of reflex syncope, is a transient loss of consciousness characterized by a sudden reduction in heart rate (HR) and blood pressure (BP) and can be associated with negative or positive head‐up tilt test (HUTT).^3^, ^4^ Diagnosis of VVS is mainly based on physical examination and careful history‐taking without the need for further neurologic examinations in typical forms of VVS^3^, ^4^; however, HUTT can be assistive in some cases. The positive HUTT is classified by VVS International Study (VASIS) criteria.^5^ These criteria categorize VVS based on BP and HR changes in HUTT which can be summarized as (1) mixed BP and HR decrease without severe bradycardia; (2A) cardioinhibitory response (BP fall before HR); (2B) severe cardioinhibitory response (HR fall before BP and/or asystole); (3) BP decrease without HR fall.

In patients with a diagnosis of VVS, the management ranges from lifestyle modifications and counterpressure maneuvers to pharmacologic prevention.^6^ Lifestyle changes are the first line of treatment in most patients.^7^ VVS triggers include but are not limited to a standing posture, heat exposure, the sight of blood, and fear.^8^ Although triggers of VVS are well‐known, there is a lack of evidence regarding predisposing factors in these patients.

Vitamin D is a key player in the pathogenesis of several diseases.^9^, ^10^ Hypovitaminosis D or vitamin D deficiency (serum 25‐hydroxyvitamin D < 50 nmol/L or <20 ng/mL) is related to prolonged hospitalization and mortality, development of chronic diseases, and worse outcomes.^11^, ^12^, ^13^ The exact link between vitamin D levels or hypovitaminosis D and VVS occurrence is still unknown. Some studies reported lower serum vitamin D and a higher rate of hypovitaminosis D in patients with VVS.^14^, ^15^ However, this association has not been yet confirmed in a pooled analysis. In this study, we compared the vitamin D levels between VVS patients and controls. Moreover, the rate of VVS was compared between patients with vitamin D deficiency and non‐vitamin D‐deficient ones.

## 2 METHODS

This systematic review and meta‐analysis was conducted in accordance with the Preferred Reporting Items for Systematic Reviews and Meta‐Analyses (PRISMA) 2020 guidelines.^16^

### 2.1 Search strategy and screening

A systematic search in international online databases including Scopus, Embase, PubMed, and Web of Science was conducted from inception to February 2023. No limitations or filters were applied in the search. Search terms were “syncope” and “vitamin D” and other related terms which are available in Supporting Information: Table 1.

Duplicate studies were removed. Two reviewers (A. K. and A. H. B.) used titles and abstracts to select appropriate studies matching predefined inclusion criteria. Afterward, full‐length articles of included studies in the first step were evaluated to find the final included studies. Finally, we evaluated the reference list of included studies.

### 2.2 Inclusion and exclusion criteria

We included any clinical studies measuring vitamin D levels in VVS patients and healthy individuals. We excluded studies without controls and studies without reporting the circulating levels of vitamin D in VVS patients and healthy individuals. Moreover, non‐English abstracts, preclinical evaluations, reviews, case reports, conference abstracts, and letters were excluded.

### 2.3 Data extraction

Independent reviewers (A. K. and A. H. B.) extracted data from each study in a predefined sheet. Extracted items were: (1) study characteristics: study design, publication year, first author's name, and location, (2) population characteristics: patients with VVS and healthy controls' population characteristics, sample size (total, VVS, and controls), mean age, and male sex percentage, and (3) vitamin D evaluations: mean serum vitamin D levels in VVS patients and healthy controls and rate of vitamin D deficiency in each group, and (4) findings of included studies.

### 2.4 Quality assessment

We used the “Newcastle‐Ottawa Quality Assessment Scale” (NOS) for observational studies to evaluate the risk of bias in included studies (A. K. and A. H. B).^17^ Three categories of bias according to NOS are selection, compatibility, and outcome. On this scale, a score of ≥7 was considered “low risk”, while 4−6 and 0−3 were regarded as “high risk” and “very high risk,” respectively.

### 2.5 Statistical analysis

We used the random‐effect meta‐analysis to compare vitamin D levels between VVS patients and controls by calculating the standardized mean difference (SMD) and its 95% confidence interval (CI). To compare the rate of VVS between vitamin D‐deficient and non‐vitamin D‐deficient individuals, we calculated the odds ratio (OR) and 95% CI using the random‐effects DerSimonian‐Laird model. As there was a zero event in one of the studies, outcome, rare event analysis by Peto's method was performed.^18^ A *p* Value of less than .05 was considered statistically significant. For the age variable, we used the formulas proposed by Luo et al.^19^ and Wan et al.^20^ to recalculate the mean and standard deviation (SD) from the median and interquartile range (IQR). Mean and SDs were merged in appropriate conditions using the Cochrane Handbook.^21^

The degree of heterogeneity between the samples was assessed based on Cochrane's Q. Higgins' I‐squared test was used to determine the heterogeneity by different percentages of variation—low: 0%−25%, moderate: 26%−75%, and high: 75%−100%. The publication bias was assessed visually by inspecting funnel plots. Moreover, Egger's and Begg's^22^, ^23^ statistical tests were used for publication bias. All analyses in this study were performed using STATA version 17.0 (Stata Corp).

## 3 RESULTS

### 3.1 Literature search and included studies

**Figure 1:**
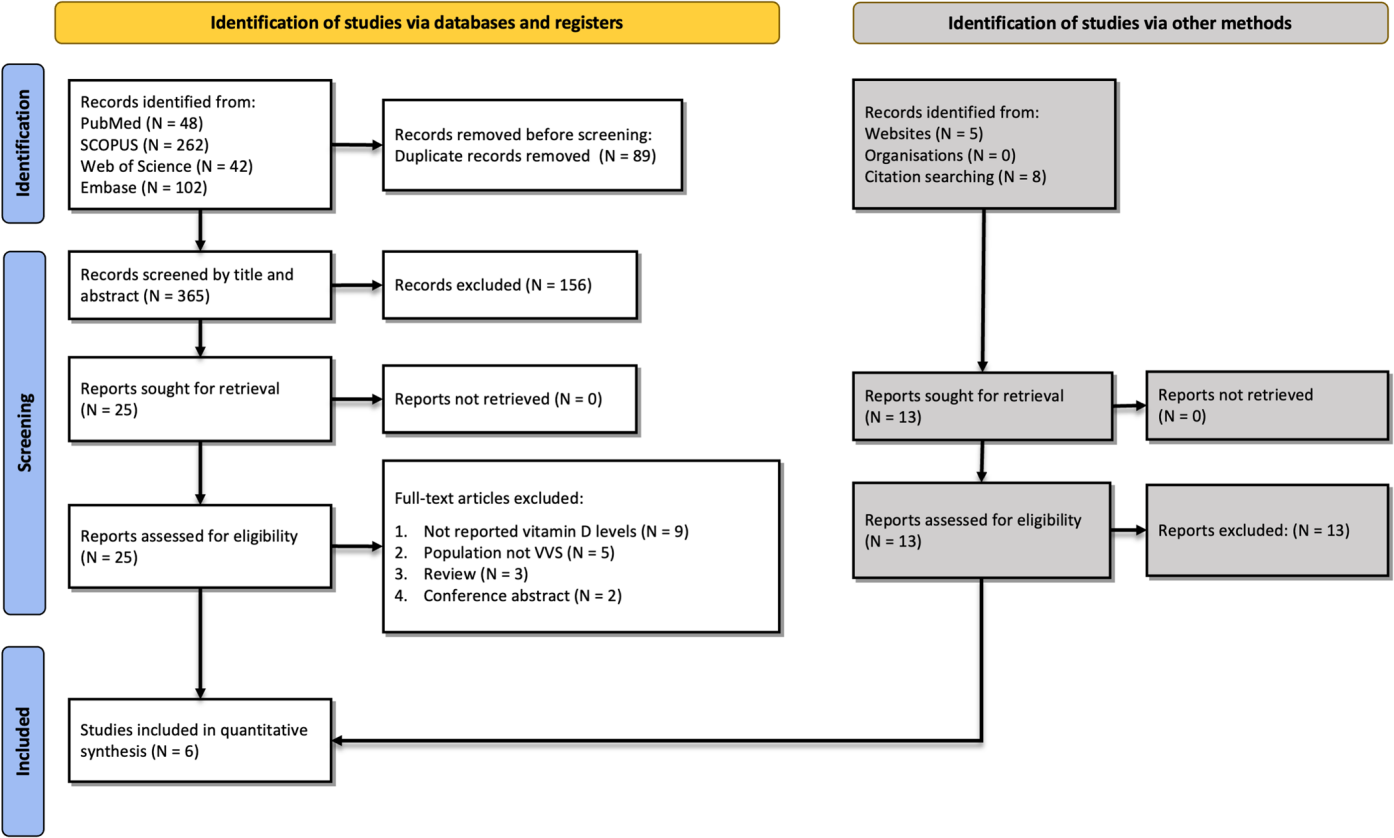
Flow diagram summarizing the selection of eligible studies based on the PRISMA guidelines. PRISMA, Preferred Reporting Items for Systematic Reviews and Meta‐Analyses.

The initial search resulted in 454 studies: PubMed (*n* = 48), Embase (*n* = 102), Web of Science (*n* = 42), and Scopus (*n* = 262). Duplicates were removed (*n* = 89) and the remaining 365 went through the first screening using titles/abstracts after which 25 studies underwent full‐text screening. Finally, six studies^14^, ^15^, ^24^, ^25^, ^26^, ^27^ remained to be included in our systematic review. Details of the search strategy and reasons for exclusion are shown in Figure 1.

Table 1 summarizes the characteristics of the studies in this review. There was a total of 954 individuals assessed in these studies. Four studies were conducted in China^15^, ^24^, ^26^, ^27^ and the design of all but one of them^24^ was a retrospective cohort. All studies were published between 2020 and 2023, and two had low risks of bias based on the NOS scoring while others had high risks of bias, details of which are represented in Supporting Information: Table 2.

**Table 1:** Baseline characteristics of included studies.

Author	Year	Design	Location	Population	*N* total	Age	% Male	Main findings
Kong et al.^24^	2022	Prospective cohort	China	Children diagnosed with VVS and healthy controls	252	NA	NA	Serum vitamin D levels were significantly lower in the VVS group compared to controls (31 ± 11 nmol/l vs. 46 ± 10 nmol/l, *p* < .001). Moreover, vitamin D deficiency was more frequent in the VVS group (73.0% vs. 24.6%, *p* < .001).
Kovalchuk et al.^14^	2023	Retrospective cohort	Ukraine	Children diagnosed with VVS and healthy controls	64	14.2 ± 2.3	56.2	VVS patients had significantly lower vitamin D levels compared to healthy individuals (18.8 ± 5.9 ng/mL vs. 30.9 ± 5.9 ng/mL). Although no control had vitamin D deficiency, 60% of patients with VVS were vitamin D deficient.
Usalp et al.^25^	2020	Retrospective cohort	Cyprus	Patients presented with VVS and healthy controls	127	32.6 ± 12.8	32.3	Vitamin D levels were significantly lower in VVS group compared to healthy individuals (20.1 ± 8.8 ng/mL vs. 24.5 ± 6.3 ng/mL, *p* = .003).
Xiao et al.^26^	2022	Retrospective cohort	China	Children with VVS and healthy controls	180	9.7 ± 2.2	52.8	Serum vitamin D levels were significantly lower in VVS patients in comparison to controls (13.07 ± 4.52 ng/mL vs. 24.56 ± 13.07 ng/mL, *p* < .0001).
Zhang et al.^15^	2021	Retrospective cohort	China	Pediatrics with VVS and healthy controls	91	11.8 ± 2.0	29.7	Levels of vitamin D were significantly lower in pediatrics with VVS compared to healthy controls (48.76 ± 19.25 nmol/l vs. 67.62 ± 15.46 nmol/l, *p* = .001). Moreover, the rate of vitamin D deficiency was significantly higher in VVS patients (60.5% vs. 13.3%, *p* = .001).
Zou et al.^27^	2021	Retrospective cohort	China	Children diagnosed with VVS and age‐ and gender‐matched healthy controls	240	10.5 ± 2.4	50.4	Children with VVS had significantly lower levels of vitamin D compared to healthy individuals (59.8 ± 21.4 nmol/l vs. 65.9 ± 19.2 nmol/l, *p* = .022). Moreover, the rate of vitamin D deficiency was significantly higher in VVS patients (33.8% vs. 20.0%, *p* = .017).

*Note*: Data are presented as mean ± standard deviation or percentage.

Abbreviations: NA, not available; VVS, vasovagal syncope.

### 3.2 Meta‐analysis

#### 3.2.1 Meta‐analysis of vitamin D levels in VVS patients

**Figure 2:**
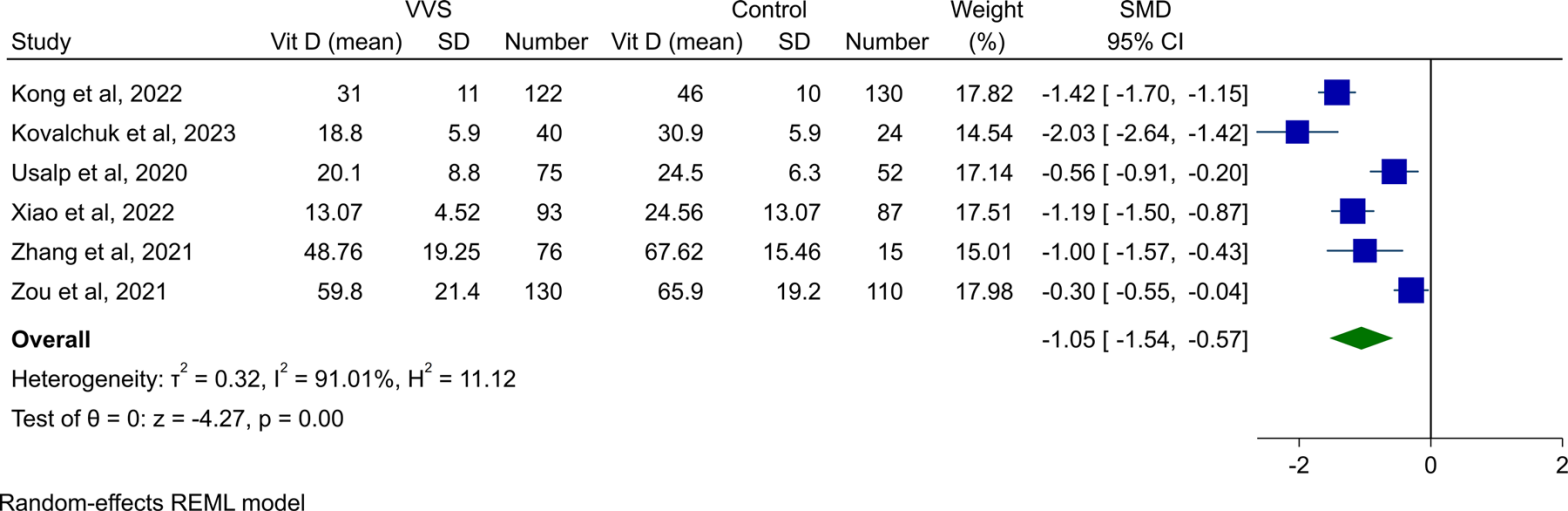
Forest plot for meta‐analysis of serum vitamin D levels in VVS patients versus healthy controls. VVS, vasovagal syncope.

Random‐effect meta‐analysis was performed for vitamin D levels in VVS patients versus controls. It was shown that vitamin D levels were lower in VVS patients (SMD −1.05, 95% CI −1.54 to −0.57, *p* < .01, Figure 2 and Table 2). The heterogeneity was found to be high (*I* ^2^: 91.01%).

**Table 2:** Outcomes of included studies.

Author (year)	*N* total	*N* VVS	*N* control	Vitamin D concentration			Rate of VVS occurrence		OR [95% CI]
				VVS	Control	SMD [95% CI]	Vitamin D‐deficient	Non‐vitamin D‐deficient	
Kong et al.^24^	252	122	130	31 ± 11	46 ± 10	−1.42 [−1.70 to −1.15]	89/121 (74%)	33/131 (25%)	6.88 [4.20−11.27]
Kovalchuk et al.^14^	64	40	24	18.8 ± 5.9	30.9 ± 5.9	−2.03 [−2.64 to −1.42]	24/24 (100%)	16/40 (40%)	12.43 [4.41−35.06]
Usalp et al.^25^	127	75	52	20.1 ± 8.8	24.5 ± 6.3	−0.56 [−0.91 to −0.20]	NA		
Xiao et al.^26^	180	93	87	13.07 ± 4.52	24.56 ± 13.07	−1.19 [−1.50 to −0.87]	NA		
Zhang et al.^15^	91	76	15	48.76 ± 19.25	67.62 ± 15.46	−1.00 [−1.57 to −0.43]	46/48 (96%)	30/43 (70%)	6.51 [2.16−19.60]
Zou et al.^27^	240	130	110	59.8 ± 21.4	65.9 ± 19.2	−0.30 [−0.55 to −0.04]	44/66 (67%)	86/174 (49%)	2.00 [1.13−3.52]

*Note*: Data are presented as mean ± standard deviation or percentage.

Abbreviations: CI, confidence interval; NA, Not applicable; OR, odds ratio; SMD, standard mean difference; VVS, vasovagal syncope.

#### 3.2.2 Meta‐analysis of VVS occurrence in vitamin D deficient versus nondeficient individuals

**Figure 3:**
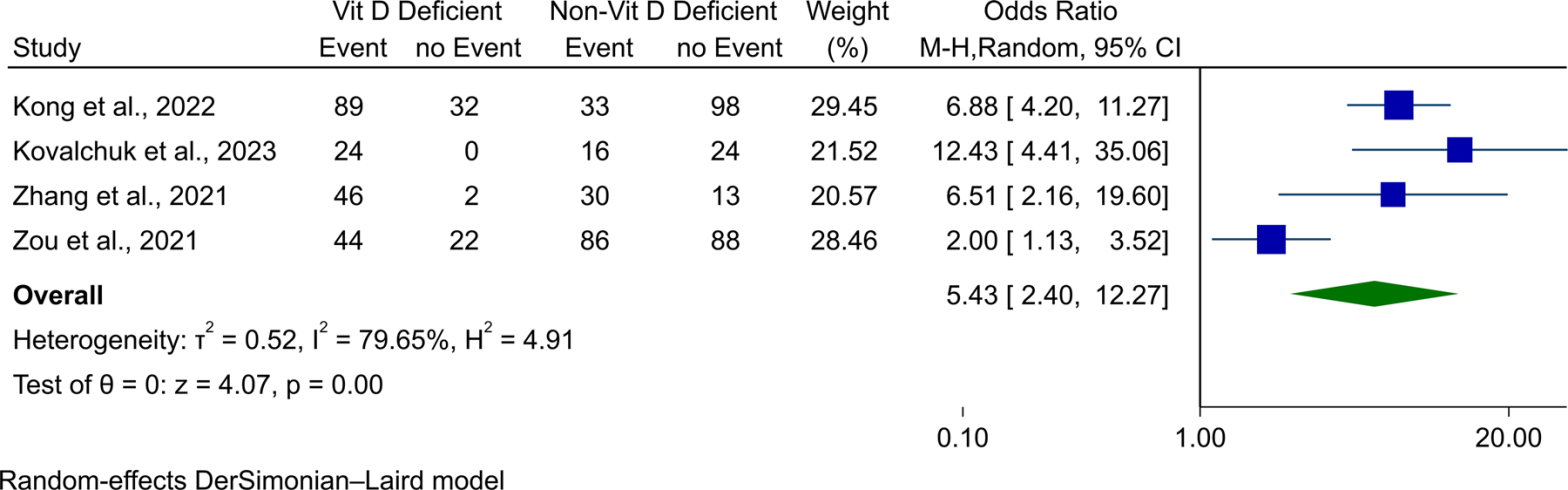
Forest plot for meta‐analysis of VVS occurrence in patients with and without vitamin D deficiency. VVS, vasovagal syncope.

Meta‐analysis of four studies to calculate OR for VVS occurrence showed that patients who are vitamin‐D deficient have higher odds of VVS, compared to those not (OR 5.43, 95% CI 2.40 to 12.27, *p*‐value < .01, Figure 3 and Table 2). We found high heterogeneity with an *I* ^*2*^ of 79.65%.

#### 3.2.3 Publication bias assessment

Funnel plots were designed for analysis of SMD for vitamin D levels and OR for VVS occurrence between individuals with vitamin D deficiency and non‐deficient cases. As shown in Supporting Information: Figure 1, no apparent asymmetry was seen in the funnel plot for the SMD meta‐analysis. Begg's and Egger's tests also did not reveal signs of publication bias (all *p* > .05). The funnel plot assessing bias for the OR is illustrated in Supporting Information: Figure 2 and as it is shown, there is an apparent asymmetry. Moreover, Begg's and Egger's analyses did not indicate significant bias (*p* = .73 and *p* = .38, respectively).

## 4 DISCUSSION

This study is the first systematic review to date assessing the association between vitamin D levels and VVS. With the inclusion of six studies, our findings implied that levels of vitamin D are significantly lower in VVS patients in comparison with healthy individuals and also there is a higher chance of VVS in vitamin D‐deficient patients. These highlight the addressing of vitamin D levels in VVS patients and also emphasize that further studies such as observational studies evaluating vitamin D levels in VVS patients and also randomized controlled trials (RCTs) to assess vitamin D supplementation in VVS patients with vitamin D deficiency are warranted.

Vitamin D deficiency is very common, with about 1 billion people suffering worldwide.^28^ In line, a study conducted on 8−18 years old children in the United States found that the prevalence of vitamin D deficiency was 77.6%.^29^ In line, it has been reported that 78.4% of Chinese children had vitamin D insufficiency.^30^ Noteworthy, five of our included studies assessed children as their population while Kovalchuk et al. found a rate of 60% deficiency ed in 40 children with VVS.^14^ The main contributing risk factors causing vitamin D deficiency are vegetarian individuals, breastfeeding or pregnant women, less exposure to sunlight, and older adults with a higher prevalence of gastrointestinal and renal disease.^25^

Vitamin D has significant roles in inflammation, the autoimmune system, and the cardiovascular system.^31^ Also, vitamin D receptors have been found in arterial wall cells,^32^ cardiomyocytes,^33^ and immune cells,^34^ all of which are major contributors to the cardiovascular system. Moreover, metabolites of vitamin D involve in cardiovascular function and disease pathways such as thrombosis, the renin‐angiotensin system, and inflammation.^35^ Similar to our findings showing lower vitamin D in VVS patients, a recent study indicated that there is a relationship between hypovitaminosis D and orthostatic hypotension.^36^

The mechanism by which hypovitaminosis D can lead to VVS is controversial. The pathophysiology by which VVS occurs is the autonomous system's abnormal reaction to triggers such as standing position which leads to the stimulation of aortic, carotid, and cardiopulmonary receptors.^37^ Moreover, a decrease in preload during VVS causes an elevation in catecholamines with VVS and consequently contraction of the empty ventricle. Activation of cardiac C fibers in turn increases vagal tone which might finally result in hypotension and bradycardia.^38^, ^39^ As mentioned, vitamin D has been shown to be involved in vascular smooth muscle cell proliferation and development, and endothelial cells as the main cells contributing to vascular elasticity. Hence, decreased 25‐[OH]‐D levels might lead to VVS by impairing vascular tone.^40^ This idea is further strengthened by the observation that Usalp et al. had by dividing patients based on the HUTT test and finding no difference in vitamin D levels.^25^

Decreased muscle function caused by low vitamin D might be another contributing reason for syncope occurrence. Mann et al. found that serum vitamin D < 20 ng/mL caused cardiac autonomic dysfunction through repression of vagal balance.^41^ Moreover, neuronal conduction disruption in the baroreflex mechanism during VVS can be affected by low vitamin D levels, as it is present in the nervous systems.^42^ In a study by Dogdus et al. a significant correlation was found between vitamin D deficiency and cardiac autonomic malfunction assessed by HR variability which was improved after supplementation with vitamin D.^43^ This was investigated in pediatric patients with VVS in studies by Zhang et al.^15^ and Zou et al.^27^ both finding significant correlations between HR variability and vitamin D levels.

The present study has clinical applications in syncope clinics in which patients with VVS are visited. Although there has been debate on the pharmacologic management of syncope, in a recent network meta‐analysis that compared all medications for VVS, only midodrine was effective in reducing syncope.^44^ Along with this and non‐pharmacologic management of VVS, vitamin D deficiency might be a missed point by clinicians. Vitamin D could be added to the regimens of those patients with VVS having vitamin D deficiency. However, RCTs are needed to address this role in the treatment of VVS.

Our study has certain limitations. First, most of our included studies' populations were children with VVS. Hence, the generalizability of our findings to the adults might be affected. In this regard, further studies focusing on adults with hypovitaminosis D are needed to elucidate this role in adults. Second, the inherent limitations of each of included studies must be taken into consideration, including not matching for confounding factors and the small number of cases assessed. Moreover, the qualities of included studies were relatively low, hence, high‐quality studies are warranted in this regard. Third, as a small number of studies were included in our meta‐analyses, we were unable to conduct meta‐regression. Finally, high heterogeneity was observed in our analyses which was partially justified by meta‐regression, in which publication year and male percentage were shown to be effective in this high heterogeneity.

## 5 CONCLUSION

This meta‐analysis compared vitamin D levels in VVS patients and healthy controls in addition to VVS occurrence in vitamin D‐deficient subjects. It was demonstrated that VVS patients had significantly lower vitamin D levels and also VVS is higher in vitamin D‐deficient cases. Although adequate studies with a powered sample size are needed to confirm this relationship and the efficacy of vitamin D in the management of VVS, this study can guide clinicians in measuring vitamin D levels routinely in VVS patients.

## AUTHOR CONTRIBUTIONS

**Amirmohammad Khalaji**: Writing—original draft/formal analysis/visualization. **Amir Hossein Behnoush**: Supervision/conceptualization/writing—review and editing. **Masih Tajdini**: Writing—review and editing. All authors read and approved the final manuscript.

## CONFLICT OF INTEREST STATEMENT

The authors declare no conflict of interest.

## Supporting information

Supporting information.

## Data Availability

The datasets generated during and/or analyzed during the current study are available from the corresponding author upon reasonable request.
