# Supplementary material for: Association between vitamin D deficiency and vasovagal syncope: A systematic review and meta‐analysis
Source: Clin Cardiol. 2023 May 24;46(7):721–8. doi: 10.1002/clc.24035 (PMC10352974; doi:10.1002/clc.24035)
Supplement: Supplementary file 1 — Supporting information. [file CLC-46-721-s001.docx]

***Supplementary Table 1.*** *The search queries used for each database and the search results*

| **Query** | | **Results (No.)** |
| --- | --- | --- |
| **PubMed** | | |
| #1 | ("syncope*"[tiab] OR "faint*"[tiab] OR “Syncope”[Mesh] OR "Syncope, Vasovagal"[Mesh]) | 33,498 |
| #2 | (“vitamin D”[tiab] OR “25(OH)D”[tiab] OR “25(OH) D”[tiab] OR “25 (OH) D”[tiab] OR “25(OH)D3”[tiab] OR “25(OH) D3”[tiab] OR “25 (OH) D3”[tiab] OR “calcifediol”[tiab] OR “calcitriol”[tiab] OR “Calciol”[tiab] OR “Vitamin D 3”[tiab] OR “Vitamin D3”[tiab] OR “Cholecalciferol*”[tiab] OR “Hydroxyvitamin D”[tiab] OR “Hydroxycholecalciferol”[tiab] OR “Calciferol*”[tiab] OR “Vitamin D 2”[tiab] OR “Vitamin D2”[tiab] OR “Ergocalciferol”[tiab] OR “25 Hydroxyvitamin D 2”[tiab] OR “25 Hydroxyergocalciferol”[tiab] OR “25-Hydroxyvitamin D2”[tiab] OR “25 Hydroxyvitamin D2”[tiab] OR “25-Hydroxycalciferol”[tiab] OR “25 Hydroxycalciferol”[tiab] OR “(3 beta,5Z,7E)-9,10-Secocholesta-5,7,10(19)-trien-3-ol”[tiab] OR “25-hydroxyvitamin D”[tiab] OR “24,25 dihydroxyvitamin D”[tiab] OR “25 hydroxyvitamin D”[tiab] OR “9,10 secocholesta 5,7,10(19) trien 23 yne 1,3,25 triol”[tiab] OR “9,10 secocholesta 5,7,10(19) trien 23 yne 3,25 diol”[tiab] OR “9,10 secocholesta 5,7,10(19),16 tetraen 23 yne 1,3,25 triol”[tiab] OR “9,10 secocholesta 5,7,10(19),22 tetraene 1,3,25,26 tetrol”[tiab] OR “9,10-Secoergosta-5,7,10(19),22-tetraene-3 beta,25-diol”[tiab] OR “Ercalcidiol”[tiab] OR “Tachystin”[tiab] OR “Dihydrotachysterin”[tiab] OR “Calcamine”[tiab]) | 88,331 |
| #3 | #1 AND #2 | 48 |
| **Embase** | | |
| #1 | ('syncope*':ab,ti,kw OR 'faint*':ab,ti,kw) | 45,848 |
| #2 | (“vitamin D”:ab,ti,kw OR “25(OH)D”:ab,ti,kw OR “25(OH) D”:ab,ti,kw OR “25 (OH) D”:ab,ti,kw OR “25(OH)D3”:ab,ti,kw OR “25(OH) D3”:ab,ti,kw OR “25 (OH) D3”:ab,ti,kw OR “calcifediol”:ab,ti,kw OR “calcitriol”:ab,ti,kw OR “Calciol”:ab,ti,kw OR “Vitamin D 3”:ab,ti,kw OR “Vitamin D3”:ab,ti,kw OR “Cholecalciferol*”:ab,ti,kw OR “Hydroxyvitamin D”:ab,ti,kw OR “Hydroxycholecalciferol”:ab,ti,kw OR “Calciferol*”:ab,ti,kw OR “Vitamin D 2”:ab,ti,kw OR “Vitamin D2”:ab,ti,kw OR “Ergocalciferol”:ab,ti,kw OR “25 Hydroxyvitamin D 2”:ab,ti,kw OR “25 Hydroxyergocalciferol”:ab,ti,kw OR “25-Hydroxyvitamin D2”:ab,ti,kw OR “25 Hydroxyvitamin D2”:ab,ti,kw OR “25-Hydroxycalciferol”:ab,ti,kw OR “25 Hydroxycalciferol”:ab,ti,kw OR “(3 beta,5Z,7E)-9,10-Secocholesta-5,7,10(19)-trien-3-ol”:ab,ti,kw OR “25-hydroxyvitamin D”:ab,ti,kw OR “24,25 dihydroxyvitamin D”:ab,ti,kw OR “25 hydroxyvitamin D”:ab,ti,kw OR “9,10 secocholesta 5,7,10(19) trien 23 yne 1,3,25 triol”:ab,ti,kw OR “9,10 secocholesta 5,7,10(19) trien 23 yne 3,25 diol”:ab,ti,kw OR “9,10 secocholesta 5,7,10(19),16 tetraen 23 yne 1,3,25 triol”:ab,ti,kw OR “9,10 secocholesta 5,7,10(19),22 tetraene 1,3,25,26 tetrol”:ab,ti,kw OR “9,10-Secoergosta-5,7,10(19),22-tetraene-3 beta,25-diol”:ab,ti,kw OR “Ercalcidiol”:ab,ti,kw OR “Tachystin”:ab,ti,kw OR “Dihydrotachysterin”:ab,ti,kw OR “Calcamine”:ab,ti,kw) | 132,564 |
| #3 | #1 AND #2 | 102 |
| **Web of Science** | | |
| #1 | (TS = "syncope*" OR TS= "faint*") | 44,490 |
| #2 | (TS = (“vitamin D”) OR TS = (“25(OH)D”) OR TS = (“25(OH) D”) OR TS = (“25 (OH) D”) OR TS = (“25(OH)D3”) OR TS = (“25(OH) D3”) OR TS = (“25 (OH) D3”) OR TS = (“calcifediol”) OR TS = (“calcitriol”) OR TS = (“Calciol”) OR TS = (“Vitamin D 3”) OR TS = (“Vitamin D3”) OR TS = (“Cholecalciferol*”) OR TS = (“Hydroxyvitamin D”) OR TS = (“Hydroxycholecalciferol”) OR TS = (“Calciferol*”) OR TS = (“Vitamin D 2”) OR TS = (“Vitamin D2”) OR TS = (“Ergocalciferol”) OR TS = (“25 Hydroxyvitamin D 2”) OR TS = (“25 Hydroxyergocalciferol”) OR TS = (“25-Hydroxyvitamin D2”) OR TS = (“25 Hydroxyvitamin D2”) OR TS = (“25-Hydroxycalciferol”) OR TS = (“25 Hydroxycalciferol”) OR TS = (“(3 beta,5Z,7E)-9,10-Secocholesta-5,7,10(19)-trien-3-ol”) OR TS = (“25-hydroxyvitamin D”) OR TS = (“24,25 dihydroxyvitamin D”) OR TS = (“25 hydroxyvitamin D”) OR TS = (“9,10 secocholesta 5,7,10(19) trien 23 yne 1,3,25 triol”) OR TS = (“9,10 secocholesta 5,7,10(19) trien 23 yne 3,25 diol”) OR TS = (“9,10 secocholesta 5,7,10(19),16 tetraen 23 yne 1,3,25 triol”) OR TS = (“9,10 secocholesta 5,7,10(19),22 tetraene 1,3,25,26 tetrol”) OR TS = (“9,10-Secoergosta-5,7,10(19),22-tetraene-3 beta,25-diol”) OR TS = (“Ercalcidiol”) OR TS = (“Tachystin”) OR TS = (“Dihydrotachysterin”) OR TS = (“Calcamine”)) | 110,501 |
| #3 | #1 AND #2 | 42 |
| **SCOPUS** | | |
| #1 | (TITLE-ABS-KEY("syncope*") OR TITLE-ABS-KEY("faint*")) | 83,861 |
| #2 | (TITLE-ABS-KEY(“vitamin D”) OR TITLE-ABS-KEY(“25(OH)D”) OR TITLE-ABS-KEY(“25(OH) D”) OR TITLE-ABS-KEY(“25 (OH) D”) OR TITLE-ABS-KEY(“25(OH)D3”) OR TITLE-ABS-KEY(“25(OH) D3”) OR TITLE-ABS-KEY(“25 (OH) D3”) OR TITLE-ABS-KEY(“calcifediol”) OR TITLE-ABS-KEY(“calcitriol”) OR TITLE-ABS-KEY(“Calciol”) OR TITLE-ABS-KEY(“Vitamin D 3”) OR TITLE-ABS-KEY(“Vitamin D3”) OR TITLE-ABS-KEY(“Cholecalciferol*”) OR TITLE-ABS-KEY(“Hydroxyvitamin D”) OR TITLE-ABS-KEY(“Hydroxycholecalciferol”) OR TITLE-ABS-KEY(“Calciferol*”) OR TITLE-ABS-KEY(“Vitamin D 2”) OR TITLE-ABS-KEY(“Vitamin D2”) OR TITLE-ABS-KEY(“Ergocalciferol”) OR TITLE-ABS-KEY(“25 Hydroxyvitamin D 2”) OR TITLE-ABS-KEY(“25 Hydroxyergocalciferol”) OR TITLE-ABS-KEY(“25-Hydroxyvitamin D2”) OR TITLE-ABS-KEY(“25 Hydroxyvitamin D2”) OR TITLE-ABS-KEY(“25-Hydroxycalciferol”) OR TITLE-ABS-KEY(“25 Hydroxycalciferol”) OR TITLE-ABS-KEY(“(3 beta,5Z,7E)-9,10-Secocholesta-5,7,10(19)-trien-3-ol”) OR TITLE-ABS-KEY(“25-hydroxyvitamin D”) OR TITLE-ABS-KEY(“24,25 dihydroxyvitamin D”) OR TITLE-ABS-KEY(“25 hydroxyvitamin D”) OR TITLE-ABS-KEY(“9,10 secocholesta 5,7,10(19) trien 23 yne 1,3,25 triol”) OR TITLE-ABS-KEY(“9,10 secocholesta 5,7,10(19) trien 23 yne 3,25 diol”) OR TITLE-ABS-KEY(“9,10 secocholesta 5,7,10(19),16 tetraen 23 yne 1,3,25 triol”) OR TITLE-ABS-KEY(“9,10 secocholesta 5,7,10(19),22 tetraene 1,3,25,26 tetrol”) OR TITLE-ABS-KEY(“9,10-Secoergosta-5,7,10(19),22-tetraene-3 beta,25-diol”) OR TITLE-ABS-KEY(“Ercalcidiol”) OR TITLE-ABS-KEY(“Tachystin”) OR TITLE-ABS-KEY(“Dihydrotachysterin”) OR TITLE-ABS-KEY(“Calcamine”)) | 166,389 |
| #3 | #1 AND #2 | 262 |
| **Total records** | | **454** |
| **Total records without duplicates** | | **365** |

***Supplementary Table 2****. Quality Assessment based on the Newcastle-Ottawa Scale (NOS) manual for cohort studies*

| **Study**  **(Year)** | **Selection** | | | | **Comparability** | **Outcome** | | | **Overall**  **Score** |
| --- | --- | --- | --- | --- | --- | --- | --- | --- | --- |
|  | **Representativeness** | **Control** | **Exposure** | **Outcome** |  | **Outcome** | **Follow-up** | **Lost to Follow-up** |  |
| Kong et al. (2022) | - | * | * | * | - | * | * | * | 6 |
| Kovalchuk et al. (2023) | - | * | * | * | - | * | * | * | 6 |
| Usalp et al. (2020) | * | * | * | * | ** | * | * | * | 9 |
| Xiao et al. (2022) | - | * | * | * | - | * | * | * | 6 |
| Zhang et al. (2021) | - | * | * | * | - | * | * | * | 6 |
| Zou et al. (2021) | - | * | * | * | ** | * | * | * | 8 |

*
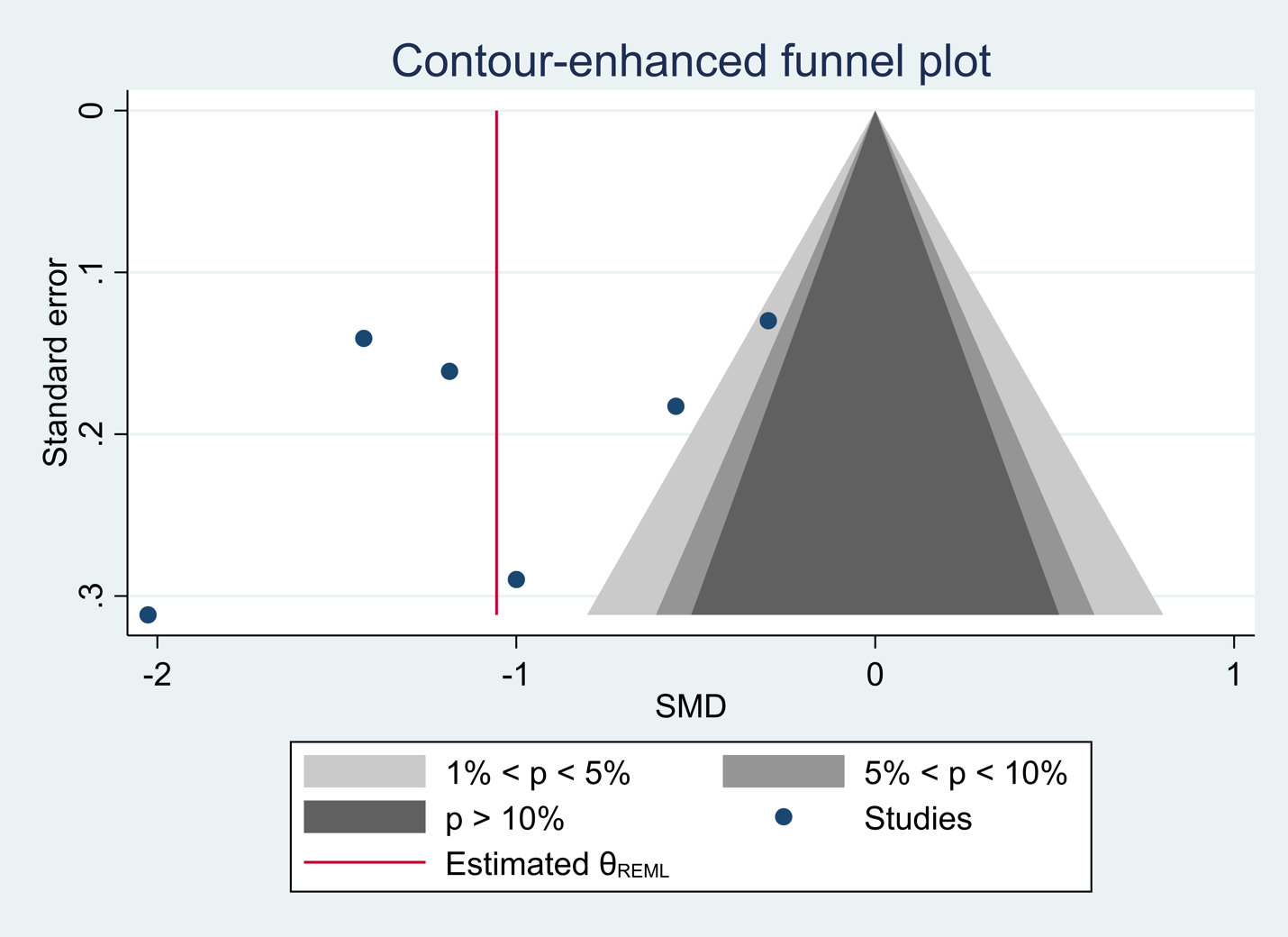
*

***Supplementary Figure 1.*** *Funnel plot for the meta-analysis of Standardized Mean Difference for Vitamin D levels between patients with vasovagal syncope vs. healthy controls*

***
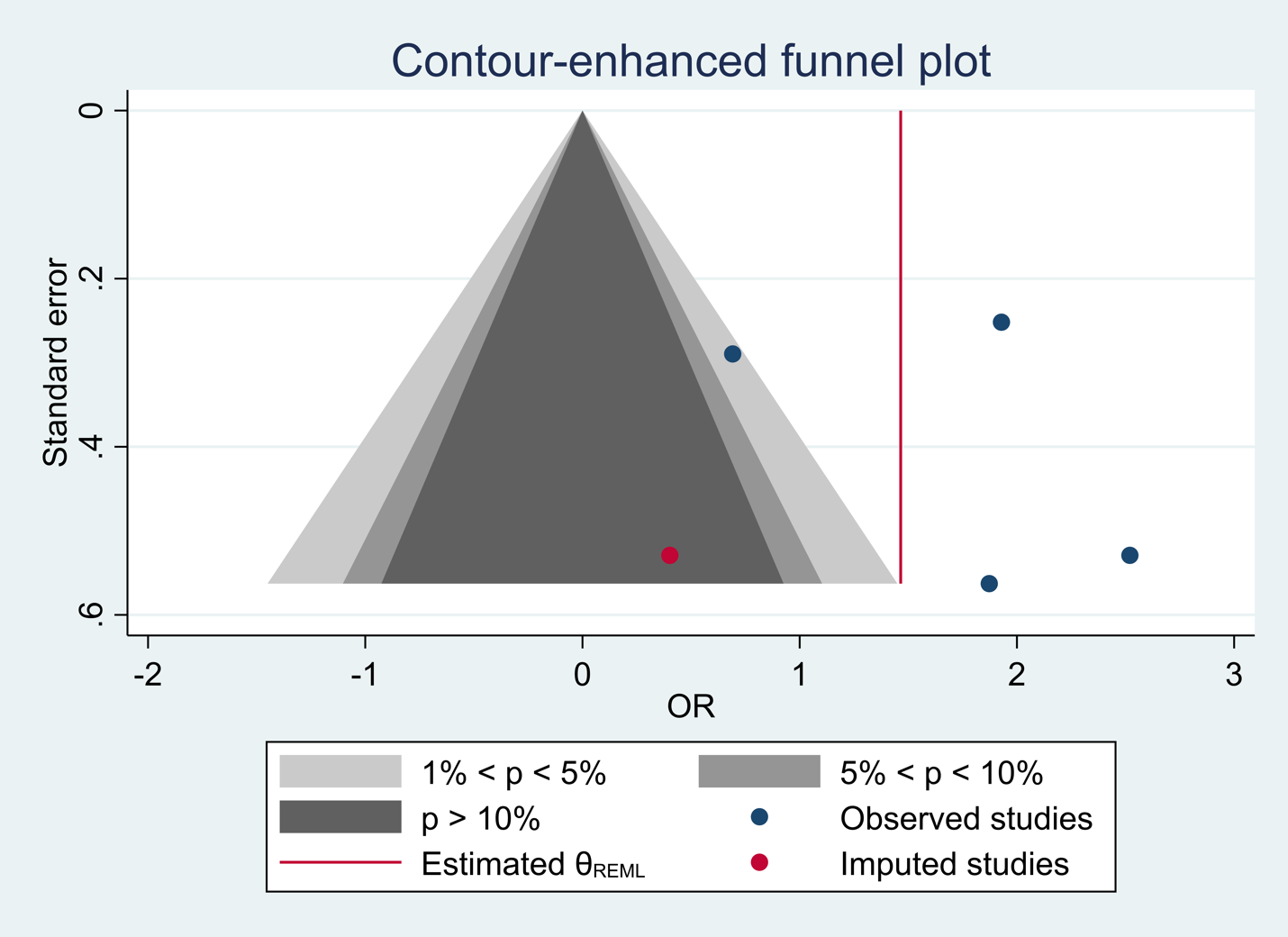
***

***Supplementary Figure 2.*** *Funnel plot for the meta-analysis of Odds Ratio for vasovagal syncope occurrence in Vitamin D deficient cases vs. non-deficient controls*
